# Health services gaps experienced by non-standard workers in Ontario, Canada: Policy implications

**DOI:** 10.1093/eurpub/ckac131.279

**Published:** 2022-10-25

**Authors:** V Gunn, P O'Campo, P Buhariwala, C Muntaner, W Lewchuk, S Baron, T Bodin

**Affiliations:** 1 Unit of Occupational Medicine, Institute of Environmental Medicine KI, Stockholm, Sweden; 2 MAP Centre for Urban Health Solutions, Li Ka Shing Knowledge Institute, Toronto, Canada; 3 Lawrence S. Bloomberg Faculty of Nursing, Dalla Lana School of Public Health, Toronto, Canada; 4 Dalla Lana School of Public Health, Toronto, Canada; 5 Department of Mental Health, The Johns Hopkins University Bloomberg School of Public Health, Baltimore, USA; 6 School of Labour Studies & Department of Economics, McMaster University, Hamilton, Canada; 7 Community Health and Social Sciences, Graduate School of Public Health and Health Policy, City University NY, New York, USA; 8 Center for Occupational and Environmental Medicine, Stockholm Region, Stockholm, Sweden

## Abstract

**Background:**

While the Canadian universal health system provides access to basic services, key health benefits are employer dependent. Given that non-standard workers (NSWs) only rarely have access to such benefits they have increased vulnerability to the many insecurities derived from their precarious employment, as clearly seen during the pandemic. The growing problem of non-standard work and workers’ heightened risk for health status deterioration, followed by a possible accentuation of health inequities, is a population health concern. This study summarizes several health services gaps experienced by NSWs and discusses policy implications and possible solutions.

**Methods:**

From January to July 2021, we conducted semi-structured interviews with a purposive sample of 40 NSWs in Ontario, Canada, part of a larger mixed-methods six-country study, including three European countries. The target population consisted of workers aged 25 to 55 who, at the time of the survey, were in non-standard employment or lost their job due to the COVID-19 pandemic.

**Results:**

Our findings highlight complex physical and mental health problems and an overall high burden of disease facing NSWs during the pandemic as linked to a combination of constant stress and worry arising from their employment insecurity, the limited and inconsistent income available to cover their basic needs, and the inadequate and unsafe working conditions they are afraid to challenge. Despite their increased health needs, given that specialized health services are not available to them for free they face financial barriers in accessing much needed health services that could help improve their health status and as a result, delay seeking care or avoid it altogether.

**Conclusions:**

Sustainable multi sectorial policy solutions are needed including the adoption of relevant labour market legislation and increases in social and health expenditures along with re-adjustments in the ways in which health services are delivered.

**Key messages:**

• During the pandemic non-standard workers in Ontario, Canada experienced complex health problems and, despite increased health needs, encountered barriers in accessing specialized health services.

• The growing problem of non-standard work and workers’ heightened risk for health status deterioration, followed by a possible accentuation of health inequities, is a population health concern.

